# A Case Report of Amyotrophic Lateral Sclerosis in a Patient With Klippel–Feil Syndrome—a Familial Occurrence: A Potential Role of TGF-β Signaling Pathway

**DOI:** 10.1097/MD.0000000000000441

**Published:** 2015-01-30

**Authors:** Zygmunt Jamrozik, Malgorzata Gawel, Katarzyna Szacka, Leopold Bakon

**Affiliations:** From the Department of Neurology (ZJ, MG, KS); and II Department of Radiology (LB), Medical University of Warsaw, Warsaw, Poland.

## Abstract

The rationale for this article is a description of a unique, familial case of a patient with amyotrophic lateral sclerosis (ALS), a progressive neurodegenerative disorder of unknown etiology coexisting with Klippel–Feil syndrome (KFS), a congenital malformation of cervical vertebrae, characterized by a fusion of minimum 2 cervical vertebrae.

We report a 68-year-old man with moderate dysarthria, fasciculations, short neck, hearing deficit, and low posterior hairline. Definite ALS was diagnosed based on neurological abnormalities and electromyography results. Magnetic resonance imaging and computed tomography showed bony abnormalities of the craniocervical junction, fusion of 2 cervical vertebrae, and syringomyelia at the level of C6-C7. KFS phenotype was noted in 2 more family members, and patient's stepsister with KFS phenotype died due to ALS.

The pedigree of our family suggests an autosomal-dominant inheritance of both syndromes. Cosegregation of ALS and KFS with an autosomal-dominant trait suggests an impairment of transforming growth factor β signaling pathway, and its potential role is discussed. Further evaluation of patients with autosomal-dominant and sporadic KFS by genetic testing, biochemical measurements, such as plasma transforming growth factor β1, and systematic follow-up electromyography seems warranted.

## INTRODUCTION

Amyotrophic lateral sclerosis (ALS) is a rare neurodegenerative disorder affecting the upper and lower motor neuron. It leads to progressive muscle weakness with atrophy. Most patients present as limb-onset ALS (70%), and the remaining ones present as bulbar-onset ALS, which usually manifests with dysarthria and/or dysphagia. Approximately, 10% of all ALS cases are familial, and the disease may be inherited in an autosomal-dominant, recessive, or X-linked way.^1^ ALS is diagnosed clinically, and the diagnosis is supported with electromyography (EMG) findings. Klippel–Feil syndrome (KFS) is characterized by fusion of cervical vertebrae, short neck, and low posterior hairline.^2,3^ Mutations in a transcription factor protein-coding mesenchyme homeobox 1 gene have been shown to cause an autosomal recessive subtype of KFS (KFS2, *Online Mendelian Inheritance in Man* (*OMIM* 214300).^4,5^ Recently, 2 types of autosomal-dominant KFS were linked to other mutations. KFS1 (OMIMs 118100) is caused by a mutation in the *Growth Differentiating Factor-6 (GDF6) gene*, whereas KFS3 (OMIM 613702) has a mutation in the GDF-3 (GDF3) gene.^6,7^ Some KFS cases are caused by chromosomal rearrangement.^8–10^

Mutations in the *Drosophila* homologue of the GDF family (GDF6) resulted in adults with morphological changes and a diminished number of spinal motor neurons and neuromuscular junctions (NMJs).^11^ Another GDF family member, GDF5, regulates dendrite growth of pyramidal cells in the hippocampus during development and survival of midbrain dopaminergic neurons.^12–14^ Thus, the role of bone morphogenetic protein (BMP)/GDF signaling is complex in the development of NMJ and survival of adult neurons including motor neurons despite its well-known role in osteogenesis and chondrogenesis.

## CLINICAL FINDINGS

The patient is a 68-year-old man with secondary education who was working as a fitter welder. His past medical history was not significant except for controlled arterial hypertension and hypercholesterolemia. In July 2012, he complained of slowly progressive dysarthria. Several months later, the patient developed generalized fasciculations. One year after the initial symptoms, the patient's speech became slurred. He was hospitalized in an ear, nose, and throat department where laryngological dysfunction was excluded.

Neurological examination showed low cognitive function (by mini mental state examination 23 score), jaw jerk, increased gag reflex, bilateral palmomental sign, pathological crying, and mild tongue atrophy with fibrillations, severe dysarthria, and hearing loss. Generalized fasciculations were observed with no muscular atrophy or muscle weakness. Tendon reflexes were increased with bilateral Jacobson sign. Sensation and coordination were normal.

The patient also showed morphological malformations including short neck and low posterior hairline.

The patient's informed consent for description of the case was given.

## DIAGNOSTIC FOCUS AND ASSESSMENT

Biochemical tests were negative, including normal serum proteins and immunoglobulin electrophoresis. Serum Lyme antibodies were negative. Cerebrospinal fluid analysis was not performed due to patient refusal. Pulmonary function testing to measure vital capacity was unfeasible due to a bulbar syndrome.

Motor nerve conduction study was performed in the right ulnar and peroneal nerves. The amplitude of compound motor action potential, distal latency, conduction velocity, and F-wave latency were found to be within the normal ranges. The parameters of sensory nerve conduction in the ulnar and sural nerves were also normal. Quantitative needle EMG was performed in the right biceps brachii, first dorsal interosseous, and tibial anterior and vastus lateral muscles. Parameters of motor unit action potentials such as amplitude, duration, and size index were found to be significantly increased in each of the examined muscles, suggesting neurogenic abnormalities such as chronic denervation (reinnervation). Spontaneous activity at rest (fasciculation potentials) was recorded in all of the above-mentioned muscles. There was no spontaneous activity in the tongue and rectus abdominal muscle. According to the revisited El Escorial criteria, clinically definite ALS was diagnosed and electrophysiological tests revealed neurogenic abnormalities suggesting neuronal degeneration in 2 segments, cervical and lumbar. According to the Awaji criteria, clinical significance of fasciculations in the presence of chronic neurogenic changes is equivalent to that of fibrillations and positive sharp waves. For this reason, the motor neurons of anterior horns at the cervical and lumbar level may be considered definitely involved in this patient.^15^

**FIGURE 1:**
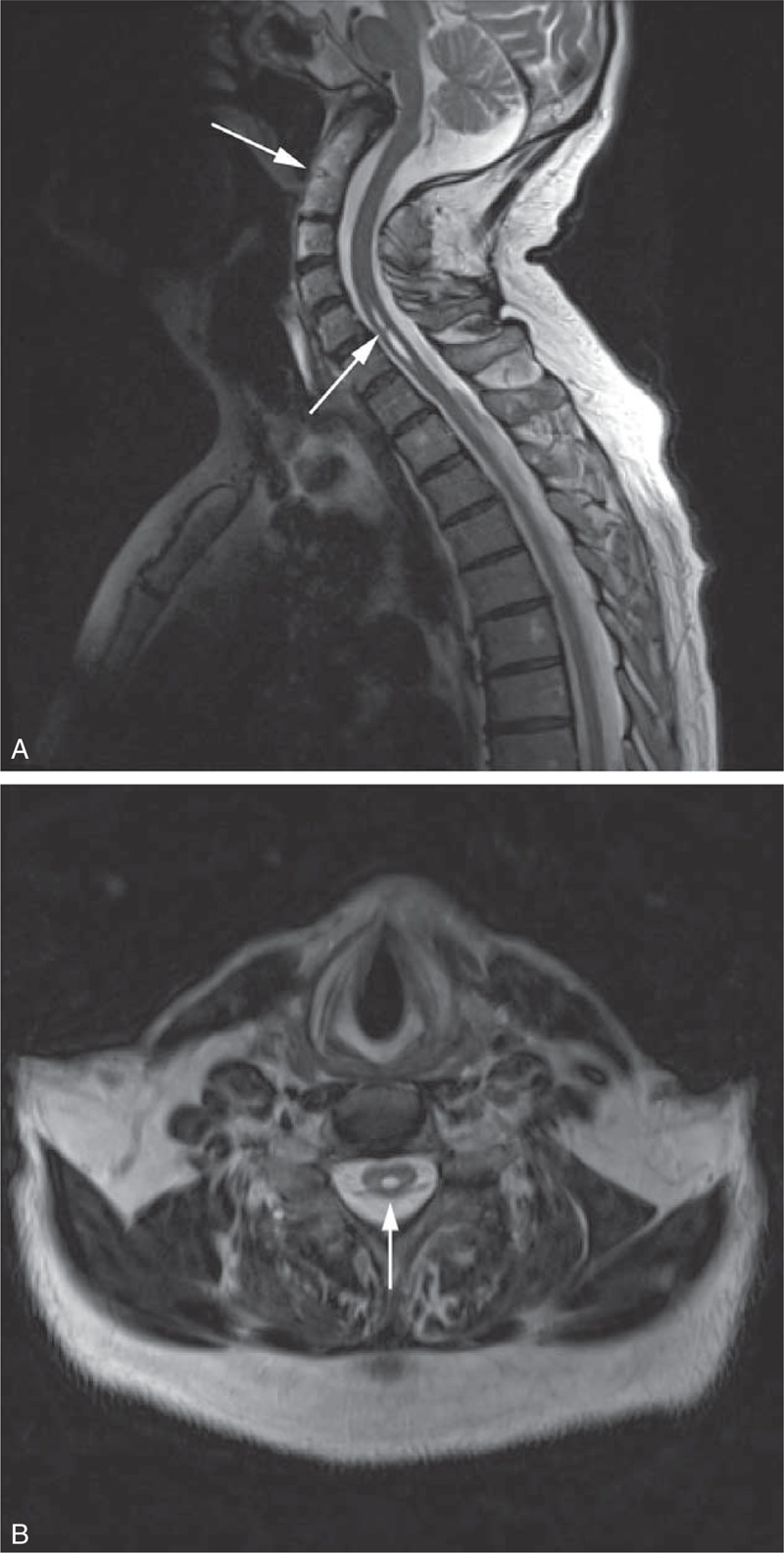
Magnetic resonance T2-weighted images. (A) Fusion of cervical vertebrae at the C2–C3 level, syringomyelia in the cervical spine (C6–C7 level). (B) Widened spinal central canal in the cervical and upper thoracic regions.

**FIGURE 2:**
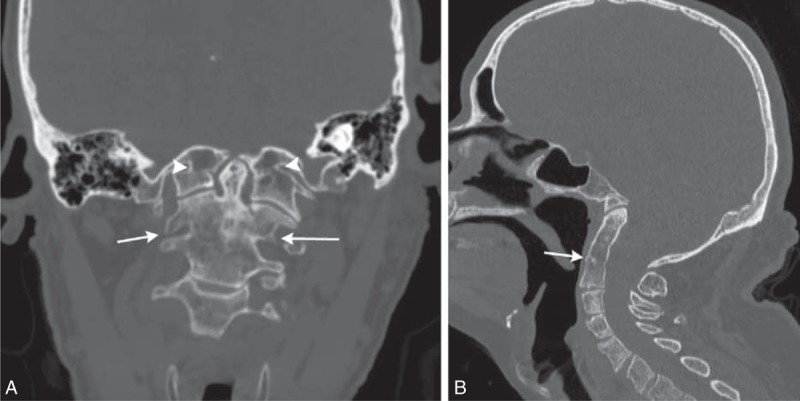
(A) Coronal reformat of CT examination. Platybasia–odontoid process is above bimastoid line. Also note atlantooccipital assimilation (arrowheads) and assimilation of C2 and C3 (arrows). (B) Sagittal reformat of CT examination. Platybasia—angle of skull base is 155 degrees, odontoid process is 25 mm above Chamberlain line. Assimilation of C2 and C3 (arrow). CT = computed tomography.

Computed tomography and magnetic resonance imaging showed bony abnormalities of the craniocervical junction, fusion of cervical vertebrae at the C2–C3 level, syringomyelia in the cervical spine (C6–C7 level), and widened central spinal canal in cervical and upper thoracic regions (Figures 1 and 2).

According to the El Escorial criteria, the diagnosis of clinically definite ALS was made. We also diagnosed KFS type II according to the Clarke classification with Chiari I malformation.^16^

**FIGURE 3:**
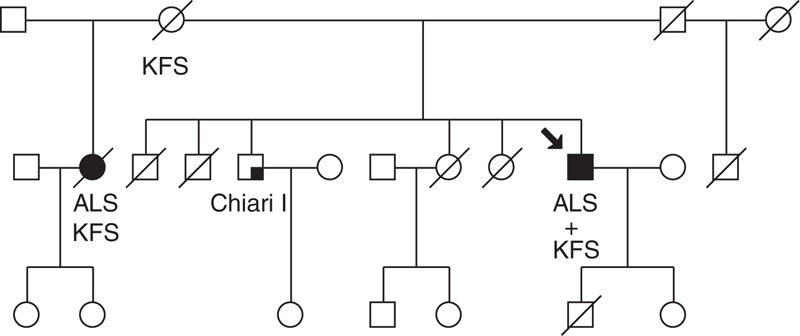
Pedigree of the reported KFS and ALS family. ALS = amyotrophic lateral sclerosis, KFS = Klippel–Feil syndrome.

The KFS phenotype was found in the patient's mother, brother, and stepsister of the same biological mother. The stepsister died at the age of 55 due to ALS (evidenced by medical records). The patient's brother has Chiari I malformation and KFS phenotype type I without vertebral fusion and with no signs of lower motor neuron involvement (Figure 3).

## DISCUSSION

We report a patient with bulbar-onset ALS and KFS with asymptomatic cervical syringomyelia. Due to the absence of sensory deficits or muscle weakness, we excluded cervical syringomyelia as responsible for the symptoms seen in our patient. The patient was asymptomatic until the bulbar dysfunction appeared, and then clinical signs of upper and lower motor neuron deficits in the cervical, thoracic, and lumbar regions developed. Clinical evaluation supported by electrophysiological and biochemical testing led to a diagnosis of ALS.

KFS is an uncommon malformation defined by a short neck, low posterior hairline, and fusion of cervical vertebrae.^2,3^ In <10% of cases, the Arnold–Chiari I malformation may be associated with craniovertebral malformations like KFS.^17^ We found 3 case reports in the literature, describing bulbar-onset ALS with Arnold–Chiari I malformation and 1 patient with KFS and ALS.^18–22^ The recently described patient with man-in-the-barrel syndrome had amyotrophy that was at least partially caused by myelopathy.^10^ Patients with KFS may have a higher risk of mechanical spinal cord injury as a consequence of unstable cervical segments. It was hypothesized that trauma and mechanical injures can implicate ALS, but recent epidemiological studies seem not to substantiate this notion. The pedigree of our family suggests an autosomal-dominant inheritance of both syndromes. To our knowledge, this is the first description of such association in a single family.

BMP-transforming growth factor β (TGF-β) signaling pathway is highly conserved in mammals and plays a role in development, angiogenesis, and stem cell proliferation. Biological activities of TGF-β are mediated by a transmembrane receptor serine/threonine complex containing 2 receptors, T*β*RI and T*β*RII. TGF-β may interact in 2 ways, through the TGF-β/activin/nodal branch via Smad 2/3, and through the BMP/GDF branch via Smad 1/5/8. GDF3 as a potential candidate for KFS may be a regulator of both TGF-β signaling branches.

Impaired BMP/TGF-β signaling was found in some neurodegenerative diseases including ALS, Huntington disease, hereditary spastic paraplegias, spinal muscular atrophy, spinobulbar muscular atrophy, and Alzheimer disease.^23^ Familial ALS and sporadic ALS also show reduced nuclear phosphorylated Smad (pSmad) levels and pSmad aggregates in cytoplasm.^24^

In case of ALS, a mutation in the vesicle-trafficking protein (VapB) has been identified in some familial cases (ALS 8). VapB mutation reduces Smad2/3, which are central mediators of TGF-β, and impairs BMP/TGF-β signaling.^25^ Another gene that influences both ALS and TGF-β pathway is zinc finger protein 512B, which codes for a transcription factor that has been established as a prognostic factor in ALS.^26^ Mice and zebrafish models of ALS suggest that the gdf6a mutation in the GDF6 gene could enhance the phenotypes of motoneuron and neuronal degeneration.^11^ A mutation in GDF6 was also found in autosomal-dominant KFS1. Regarding phenotype-gene relationships, our family belongs to KFS syndrome 1 (phenotype MIM #118100, gene #601147). Two sporadic cases of KFS and ALS reported by Umamaheshwar et al^27^ raise a possibility that coexistence of these pathologies is not that rare. Despite lack of genetic testing in our family, cosegregation of ALS and KFS with an autosomal-dominant trait strongly suggests an impairment of TGF-β signaling pathway. Further evaluation of patients with autosomal-dominant and sporadic KFS by genetic testing, biochemical measurements, such as plasma TGF-β1, and systematic follow-up EMG seems warranted.
